# Development and Validation of a Machine Learning Individualized Treatment Rule in First-Episode Schizophrenia

**DOI:** 10.1001/jamanetworkopen.2019.21660

**Published:** 2020-02-21

**Authors:** Chi-Shin Wu, Alex R. Luedtke, Ekaterina Sadikova, Hui-Ju Tsai, Shih-Cheng Liao, Chen-Chung Liu, Susan Shur-Fen Gau, Tyler J. VanderWeele, Ronald C. Kessler

**Affiliations:** 1 Department of Psychiatry, National Taiwan University Hospital & College of Medicine, National Taiwan University, Taipei City, Taiwan; 2 Department of Statistics, University of Washington, Seattle; 3 Vaccine and Infectious Disease Division, Fred Hutchinson Cancer Research Center, Seattle, Washington; 4 Department of Health Care Policy, Harvard Medical School, Boston, Massachusetts; 5 Department of Epidemiology, Harvard T.H. Chan School of Public Health, Boston, Massachusetts; 6 Institute of Population Health Sciences, National Health Research Institutes, Zhunan, Taiwan; 7 Department of Biostatistics, Harvard T.H. Chan School of Public Health, Boston, Massachusetts

## Abstract

**Importance:**

Little guidance exists to date on how to select antipsychotic medications for patients with first-episode schizophrenia.

**Objective:**

To develop a preliminary individualized treatment rule (ITR) for patients with first-episode schizophrenia.

**Design, Setting, and Participants:**

This prognostic study obtained data from Taiwan's National Health Insurance Research Database on patients with prescribed antipsychotic medications, ambulatory claims, or discharge diagnoses of a schizophrenic disorder between January 1, 2005, and December 31, 2011. An ITR was developed by applying a targeted minimum loss–based ensemble machine learning method to predict treatment success from baseline clinical and demographic data in a 70% training sample. The model was validated in the remaining 30% of the sample. The probability of treatment success was estimated for each medication for each patient under the model. The analysis was conducted between July 16, 2018, and July 15, 2019.

**Exposures:**

Fifteen different antipsychotic medications.

**Main Outcomes and Measures:**

Treatment success was defined as not switching medication and not being hospitalized for 12 months.

**Results:**

Among the 32 277 patients in the analysis, the mean (SD) age was 36.7 (14.3) years, and 15 752 (48.8%) were male. In the validation sample, the treatment success rate (SE) was 51.7% (1.0%) under the ITR and was 44.5% (0.5%) in the observed population (*Z* = 7.1; *P* < .001). The estimated treatment success if all patients were given a prescription for 1 medication was significantly lower for each of the 13 medications than under the ITR (*Z* = 4.2-16.8; all *P* < .001). Aripiprazole (3088 [31.9%]) and amisulpride (2920 [30.2%]) were the medications most often recommended by the ITR. Only 1054 patients (10.9%) received ITR-recommended medications. Observed treatment success, although lower than the success under the ITR, was nonetheless significantly higher than if medications had been randomized (44.5% [SE, 0.55%] vs 41.3% [SE, 0.4%]; *Z* = 6.9; *P* < .001), although only marginally higher than if medications had been randomized in their observed population proportions (44.5% [SE, 0.5%] vs 43.5% [SE, 0.4%]; *Z* = 2.2; *P* = .03]).

**Conclusions and Relevance:**

These results suggest that an ITR may be associatded with an increase in the treatment success rate among patients with first-episode schizophrenia, but experimental evaluation is needed to confirm this possibility. If confirmed, model refinement that investigates biomarkers, clinical observations, and patient reports as additional predictors in iterative pragmatic trials would be needed before clinical implementation.

## Key Points

**Question:**

Can an individualized treatment rule be developed from administrative data to improve antipsychotic medication selection for patients with first-episode schizophrenia?

**Findings:**

In this prognostic study of the records of 32 277 Taiwanese patients with first-episode schizophrenia, an individualized treatment rule based on an ensemble machine learning method was developed. Using this rule, which was developed in a 70% training sample, the estimated treatment success rate was 51.7% when the rule was applied to the remaining 30% of patients in the holdout sample, which was significantly higher than the observed 44.5% treatment success rate in the holdout sample.

**Meaning:**

Results of this study suggest that a clinically useful individualized treatment rule for antipsychotic medication selection could be associated with an increase in the treatment success in first-episode schizophrenia, although a clinical trial and replication of this result with an expanded predictor set would be needed before clinical implementation.

## Teaser

This prognostic study describes the development and validation of an individualized treatment rule for patients with first-episode schizophrenia.

## Introduction

Schizophrenia is a severe and persistent mental disorder.^1,2^ The standard treatment of antipsychotic medication reduces symptoms and relapse.^3^ However, a study found that only 60% of patients with first-episode schizophrenia achieved significant symptom reduction with their first antipsychotic medication and that 33% to 72% of cases changed medications or discontinued treatment in the 12 months after initiation because of insufficient efficacy or adverse effects.^4^ Inadequate treatment increases risk of both relapse^5^ and poor long-term functional outcomes.^6^

Numerous clinical trials and observational studies have investigated comparative treatment effects and risk profiles of antipsychotic drugs.^3,7,8^ Others have searched for reliable prognostic predictors of antipsychotic treatment response (ie, factors associated with treatment response regardless of treatment type) to help guide the decisions about adjunctive psychosocial treatment and psychoeducation,^9^ while a small number of recent studies have searched for reliable prescriptive predictors of antipsychotic treatment response (ie, predictors of which antipsychotic medications are best for which patients).^10,11^ The prescriptive predictors found so far have been too weak to be of clinical value individually,^12,13^ leading to a focus on composite models to develop individualized treatment rules (ITRs).^14,15^ Although promising results have been reported in ITR development,^14,15^ no practical ITR has yet been proposed. In the absence of such information, treatment decisions must rely on considerations of pharmacokinetics and pharmacodynamics and trial-and-error investigations of tolerability.^16^

Two barriers exist to developing antipsychotic ITRs. First, much larger samples are needed than in previous trials^17,18,19^ to achieve adequate statistical power.^20^ Second, more sophisticated statistical methods are needed than used in other studies.^21^ We addressed these problems by carrying out a proof-of-concept study in which we developed a preliminary ITR for initial antipsychotic drug selection in first-episode schizophrenia. This ITR was preliminary because it was based on an observational design rather than a randomized clinical trial design and because it used a less extensive set of predictors than ideal, but the exercise nonetheless demonstrated the potential value of our approach.

We considered an observational sample of more than 30 000 patients who were treated as outpatients for first-episode schizophrenia. Although less able than controlled trials to balance confounding variables across treatments, efforts to approximate the balance obtained in controlled trials by adjusting for imbalance in observed covariates often yield comparative effectiveness estimates similar to those obtained in randomized trials.^22,23^ Extensions are available to develop ITRs,^24,25,26^ which does not mean that such results are definitive; they are not. However, as noted in a recent expert panel review of best practices in studying treatment effect heterogeneity,^27^ applying an ITR developed in a previous observational study to patient-level data in a controlled trial can sometimes be more useful than attempting to both develop and evaluate the ITR in a trial. When observational data are used in this way to develop an ITR, however, it is critical to recognize that this ITR can be considered no more than preliminary until it is evaluated in 1 or more clinical trials.

Potential prescriptive predictors are evaluated conventionally in controlled trials and observational studies by computing the interactions between predictors and dummy variables for treatment type to estimate treatment response.^28^ When multiple significant interactions are present, a composite ITR is created by computing predicted outcome scores for each patient under each treatment regimen based on the model and comparing medication-specific patient-level predicted outcome scores to select the medication with the best such score for each patient.^29^ The accuracy of this approach, however, requires correct specification of both the (possibly nonlinear) associations of individual predictors with the outcome and the (possibly complex nonlinear and higher-order) interactions among predictors. We used a machine learning method to address this problem by estimating interactions directly without requiring the correct specification of associations of individual predictors with the outcome and using a flexible ensemble learning approach to improve the accuracy of specifying interactions. The advantage of this approach over the conventional method of developing ITRs applies equally to investigations based on controlled trials and those based on observational studies.

## Methods

### Data Source

Data were obtained from Taiwan's National Health Insurance Research Database, which contains all reimbursed claims of Taiwan National Health Insurance, a mandatory government-sponsored single-payer health insurance program that covers 99% of the 23 million citizens of Taiwan. This database stores data on demographic characteristics, clinical diagnoses, procedures, and prescription claims and has previously been used to study schizophrenia^30^ and comparative effectiveness of antipsychotic drugs.^31,32^ The study protocol was approved by the Research Ethics Committee of National Taiwan University Hospital, which provided a waiver of informed consent because the data used were deidentified. This study followed the Transparent Reporting of a Multivariable Prediction Model for Individual Prognosis or Diagnosis (TRIPOD) reporting guideline. It was conducted between July 16, 2018, and July 15, 2019.

Records were extracted for all patients with 1 or more antipsychotic medications prescribed by a psychiatrist and with either 3 or more ambulatory claims or 1 or more inpatient discharge diagnoses of a schizophrenic disorder (*International Classification of Diseases, Ninth Revision, Clinical Modification,* code 295.x) between January 1, 2005, and December 31, 2011 (eFigure in the Supplement). Only incident cases with first antipsychotic prescription between 2005 and 2011 were retained. Additional patients were excluded if they were outside the age range of 16 and 74 years or if their first antipsychotic medication was a second-line regimen (eg, clozapine, polypharmacy, or long-acting injectables, which were not commonly used for incident cases in Taiwan over the study period). Because details of inpatient treatments were unavailable, patients who received their first antipsychotic prescription during hospitalization were excluded. Patients treated with rarely prescribed antipsychotic drugs were excluded. The remaining patients were the focus of this prognostic study.

### Measures

#### Antipsychotic Medications

We defined antipsychotic medications (code N05A) according to the Anatomical Therapeutic Chemical Classification System.^33^ The 15 antipsychotic drugs included in this study were amisulpride, aripiprazole, chlorpromazine hydrochloride, clothiapine, flupentixol, haloperidol, olanzapine, paliperidone, quetiapine fumarate, risperidone, sulpiride, thioridazine hydrochloride, trifluoperazine hydrochloride, ziprasidone hydrochloride, and zotepine.

#### Treatment Outcome

The primary measure of treatment success was defined as the absence for 12 months of inpatient mortality and either hospitalization for any reason or change in treatment (ie, switch to or addition of another antipsychotic medication). Consistent with previous studies,^8,34^ we included hospitalization for any reason, because schizophrenia is associated with substantially elevated risk of numerous physical and emotional health problems, although we were unable to assess the emergence of chronic conditions that did not require hospitalization. Change in medication was included, because it indicates that the initial treatment was inadequate. We did not include discontinuation of treatment as an indicator of treatment failure, however, because a patient might have a good response and still discontinue therapy. We also did not include dose change as an indicator because titration is often used to maintain good response while avoiding adverse effects. However, we conducted sensitivity analyses using alternative definitions of treatment success to investigate the implications of these decisions.

#### Predictors

Patient demographic and clinical characteristics, including medical and psychiatric comorbid conditions, were identified from claims records in the 12 months up to the index date. In addition, information was extracted about current (at the index date) and past use of psychotropic medications and other drugs to treat medical comorbidities associated with psychiatric conditions. Overall, 121 variables were included in the analysis (eTable 1 in the Supplement).

### Statistical Analysis 

#### Estimating the Preliminary ITR

We estimated the ITR in a 70% random (training) sample of the patients (n = 22 601) with the *sg* R package^24,35,36^ (R Project for Statistical Computing) to obtain patient-level estimates of the differences in probability of treatment success under each medication compared with success probability in the observed population. The treatment with the highest patient-level–predicted probability of success was the one selected by the ITR for that patient. First, *sg* used the Super Learner, a targeted minimum loss–based ensemble machine learning method, to estimate the propensity of receiving a given medication and to generate a preliminary predicted probability of treatment success under each medication and in the observed population for each patient adjusting for baseline propensities.^37^ The Super Learner model uses 10-fold cross-validation to select a combination of weights across a collection (ensemble) of candidate classifiers to combine predicted probabilities of treatment success in such a way as to guarantee prediction accuracy that is at least as good as that of the best component classifier according to a prespecified criterion (which we specified to be the minimizing mean-squared prediction error). Consistent with recommendations,^38^ we used diverse candidate algorithms to optimize Super Learner performance (eTable 2 in the Supplement).

After patient-level, medication-specific probabilities of treatment success were estimated, they were subtracted from the patient-level probability estimated for the total population. These approximately unbiased estimates of probability difference were used as outcomes in a second series of 15 Super Learner models that estimated interactions (ie, within-patient difference scores) directly. As noted, this approach has the advantage of not needing estimates of the associations between individual predictors and the outcomes. Furthermore, the model improves on other approaches that estimate interactions directly by using an ensemble of classifiers rather than relying on any single classifier to specify the interactions correctly.^24^

#### Evaluating ITR Performance

The aggregate outcome of the ITR was quantified using a targeted minimum loss–based estimator of attained improvement in the outcome based on ITR-recommended treatment compared with alternative schemes,^39^ as implemented in the *tmle* R package.^40^ The *tmle* package adjusts for measured confounders using a doubly robust approach that leverages both the estimate of the propensity score and the estimate of the predicted probability of success given the treatment and covariates. Validation of the ITR was carried out in the 30% of the sample (n = 9676) not involved in estimating the ITR. Alternative treatment rules were also evaluated, including the observed treatment allocation, randomization of patients across all 15 medications (ie, one-fifteenth of patients randomized to each medication), randomization using each medication in the proportion it was actually prescribed, and 15 schemes in which all patients were assigned to only 1 medication. Statistical evaluations of differences in estimated treatment success rates were carried out using 1-sample *t* tests with a 1-sided significance level of .05.

#### Examining Key Prescriptive Predictors

We examined the relative importance of predictors in estimating individual-level predicted difference scores in each of the 15 final models. We used the random forests variable importance method of permuting 1 predictor at a time to compare increases in mean-squared error.^41^

## Results

In total, 32 277 patients were included in the present analysis. Of these patients, the mean (SD) age was 36.7 (14.3) years, and 15 752 (48.8%) were male. See eTable 1 in the Supplement for a detailed description of other patient characteristics.

### Observed and ITR-Recommended Distributions of Antipsychotic Medications

Risperidone (distributed to 6743 patients [29.8%] in the training sample and 2905 [30.0%] in the validation sample), sulpiride (6455 [18.6%] in the training sample and 2747 [28.4%] in the validation sample), and quetiapine (2274 [10.1%] in the training sample and 1004 [10.4%] in the validation sample) were the most commonly prescribed medications, but they were seldom recommended by the ITR for patients in the validation sample (risperidone, 1069 [11.1%]; sulpiride, 105 [1.1%]; and quetiapine, 385 [4.0%]) (Table 1). Aripiprazole (3088 [31.9%]) and amisulpride (2920 [30.2%]) were the most commonly ITR-recommended medications for patients in the validation sample, but they were seldom prescribed (aripiprazole, 614 [6.3%]; amisulpride, 674 [7.0%]).

**Table 1.** Observed vs Recommended Distributions of Prescribed Medications

Prescribed Medication	Observed Distribution^a^				ITR-Recommended Distribution, Validation Sample	
	Training Sample		Validation Sample			
	% (SE)	No.	% (SE)	No.	% (SE)	No.
Amisulpride	6.9 (0.6)	1552	7.0 (1.0)	674	30.2 (0.8)	2920
Aripiprazole	6.0 (0.6)	1353	6.3 (1.0)	614	31.9 (0.8)	3088
Chlorpromazine hydrochloride	1.0 (0.7)	231	1.1 (1.0)	103	1.2 (1.0)	119
Clothiapine	0.7 (0.7)	149	0.5 (1.0)	47	0.02 (1.0)	2
Flupentixol	1.7 (0.7)	381	1.6 (1.0)	158	0.6 (1.0)	55
Haloperidol	5.5 (0.6)	1235	5.2 (1.0)	503	0.6 (1.0)	62
Olanzapine	4.6 (0.6)	1040	4.5 (1.0)	433	5.0 (1.0)	479
Paliperidone	1.2 (0.7)	271	1.2 (1.0)	116	8.6 (1.0)	831
Quetiapine fumarate	10.1 (0.6)	2274	10.4 (1.0)	1004	4.0 (1.0)	385
Risperidone	29.8 (0.6)	6743	30.0 (0.9)	2905	11.1 (1.0)	1069
Sulpiride	18.6 (0.5)	6455	28.4 (0.9)	2747	1.1 (1.0)	105
Thioridazine hydrochloride	0.4 (0.6)	99	0.3 (1.0)	28	0	0
Trifluoperazine hydrochloride	1.1 (0.7)	243	1.1 (1.0)	106	8.5 (1.0)	339
Ziprasidone hydrochloride	1.1 (0.7)	238	1.0 (1.0)	101	0	0
Zotepine	1.5 (0.7)	337	1.4 (1.0)	137	2.3 (1.0)	222
Total No.	NA	22 601	NA	9676	NA	9676

Abbreviations: ITR, individualized treatment rule; NA, not applicable.

^a^ Observed distributions of prescribed medications did not differ between the 2 samples (χ^2^_14_ = 11.2; *P* = .67).

Only 1054 of 9676 patients (10.9%) in the 30% holdout sample received ITR-recommended medications. The proportion of patients with prescribed ITR-recommended medication varied significantly across medications owing to the differences in proportional use (χ^2^_14_ = 903.6; *P* < .001) (Table 2). This confounding factor was removed by calculating the proportion of times a given medication was prescribed to patients for whom it was ITR recommended divided by the same proportion when the medication was not recommended. This ratio varied significantly across medications (χ^2^_14_ = 87.0; *P* < .001). It was 0 for the 5 least-prescribed medications (chlorpromazine, clothapine, flupentixol, thioridazine, and ziprasidone), not significantly different from 1.0 for 5 others (haloperidol, 0.5 [95% CI, 0.0-1.4]; olanzapine, 0.7 [95% CI, 0.5-1.1]; paliperidone, 0.7 [95% CI, 0.3-1.2]; sulpride, 1.0 [95% CI, 0.7-1.2]; and trifluoperazine, 1.7 [95% CI, 0.6-2.9]), significantly greater than 1.0 but modestly elevated for 3 others (amisulpride, 1.2 [95% CI, 1.0-1.3]; aripiprazole, 1.3 [95% CI, 1.2-1.5]; and risperidone, 1.4 [95% CI, 1.3-1.5]), and substantially elevated for only 2 (quetiapine, 2.7 [95% CI, 2.3-3.1]; zotepine, 3.0 [95% CI, 1.4-4.8]).

**Table 2.** Proportions of Patients in the Validation Sample With Prescribed Recommended vs Nonrecommended Medication

Medication	Proportion of Patients, % (SE)		Estimated Ratio of Prescribed ITR-Recommended Medication to Prescribed Nonrecommended Medication (95% CI)^b^
	With Prescribed ITR-Recommended Medication^a^	With Prescribed Nonrecommended Medication	
Amilsupride	7.8 (0.5)	6.6 (0.3)	1.2 (1.0-1.3)
Aripiprazole	7.6 (0.5)	5.8 (0.3)	1.3 (1.2-1.5)
Chlorpromazine hydrochloride	0	1.1 (0.1)	0
Clothapine	0	0.5 (0.1)	0
Flupentixol	0	1.6 (0.1)	0
Haloperidol	3.2 (2.3)	5.2 (0.2)	0.6 (0.0-1.4)
Olanzapine	3.3 (0.8)	4.5 (0.2)	0.7 (0.5-1.1)
Paliperidone	0.8 (0.3)	1.2 (0.1)	0.7 (0.3-1.2)
Quetiapine fumarate	26.2 (2.2)	9.7 (0.3)	2.7 (2.3-3.1)
Risperidone	39.6 (1.5)	28.8 (0.5)	1.4 (1.3-1.5)
Sulpride	27.6 (5.0)	28.4 (0.5)	1.0 (0.7-1.2)
Thioridazine hydrochloride	0	0	0
Trifluoperazine hydrochloride	1.8 (0.7)	1.1 (0.1)	1.7 (0.6-2.9)
Ziprasidone hydrochloride	0	0	0
Zotepine	4.1 (1.3)	1.4 (0.1)	3.0 (1.4-4.8)

Abbreviation: ITR, individualized treatment rule.

^a^ Proportion of patients varied significantly across medications (χ^2^_14_ = 903.6; *P* < .001).

^b^ Ratios varied significantly across medications (χ^2^_14_ = 87.0; *P* < .001).

### Estimated Aggregate Treatment Success of the ITR 

The estimated aggregate treatment success rate under the ITR was 51.7% (SE, 1.0%), representing a 16% proportional increase over the 44.5% (SE, 0.5%) treatment success rate in the observed population (ie, [51.7-44.5] / 44.5 = 1.16; *Z* = 7.1; *P* < .001) and corresponding to a number needed to treat of 13.9. Sensitivity analyses showed that this proportional increase would have been unchanged if treatment failure were defined more narrowly to include either psychiatric hospitalization or treatment change ([54.0-46.4] / 46.4 = 1.16; *Z* = 9.6; *P* < .001), would have remained significant if treatment failure were defined exclusively as psychiatric hospitalization ([86.9-81.7] / 81.7 = 1.06; *Z* = 9.2; *P* < .001) or treatment change ([57.0-49.5] / 49.5 = 1.15; *Z* = 9.5; *P* < .001), and would have increased from 16% to between 40% and 48%, with the number needed to treat decreasing to 11.1, if the definition of treatment failure were expanded to include discontinuation within 3 to 9 months (eTable 3 in the Supplement).

The observed treatment success rate, although significantly lower than under the ITR, was significantly higher than if medications were randomized with equal probability; that is, if one-fifteenth of patients were randomized to 1 of the 15 medications (44.5% [SE, 0.5%] vs 41.3% [SE, 0.4%]; *Z* = 6.9; *P* < .001). This result suggested that clinicians were aware that some medications were more effective than others. However, the observed treatment success rate was only slightly higher than if patients were randomized to receive medications in the proportions actually prescribed, that is, if 6.9% of patients were randomized to amisulpride, 10.1% to quetiapine, or 29.8% to risperidone (44.5% [SE, 0.5%] vs 43.5% [SE, 0.4%]; *Z* = 2.2; *P* = .03). This result suggested that clinicians were not much better than chance in identifying which patients should be given a prescription for which medication. This finding is consistent with the prescribing ratios in Table 2.

We also estimated treatment success rates under scenarios in which all patients received a prescription for a single medication, although such estimates could be done only for 13 of the medications because the other 2 (clothiapine and thioridazine) were prescribed to too few patients (0.3%-0.5% of patients). The estimated aggregate treatment success rate under the ITR (51.7%) was significantly higher than in each of these 13 comparison scenarios (*Z* = 4.2-16.8; all *P* < .001) (Table 3). The estimated medication-specific treatment success rates were all relatively comparable to the observed rates among patients with a prescription for these medications (eg, 45.0% [SE, 1.9%] of those who received amilsulpride had treatment success compared with the estimated 45.4% [SE, 1.9%] treatment success if all in the validation sample received amilsulpride), a finding consistent with the observation that clinicians are not much better than chance in matching patients to medications. Medication-specific treatment success rates were much higher, in comparison, among patients receiving ITR-recommended medications (eg, if those who received amilsulpride instead received the medication recommended by the ITR, we would expect to see an estimated 6.3% [SE, 2.1%] more patients with treatment success).

**Table 3.** Observed and Estimated Medication-Specific Treatment Success Rates for Each Recommended Medication

Medication	Observed Success Rate in the Total Validation Sample		Estimated Success Rate in the Total Validation Sample if All Patients Received the Medication, % (SE)	Estimated Difference in Success Rate Between Patients With ITR-Recommended Medication and if All Patients Received the Medication, % (SE)^b^	Estimated Success Rate Only for Patients for Whom the Medication Was ITR Recommended, % (SE)^c^
	% (SE)	No.^a^			
Amisulpride	45.0 (1.9)	303	45.4 (1.9)	6.3 (2.1)^d^	50.9 (2.0)
Aripiprazole	45.1 (2.0)	277	45.7 (2.0)	6.0 (2.2)^d^	51.3 (1.7)
Chlorpromazine hydrochloride	36.9 (4.8)	38	36.6 (1.4)	15.2 (1.7)^d^	45.7 (4.6)
Clothiapine	27.7 (6.5)	13	NA^e^	NA^e^	58.1 (55.2)
Flupentixol	41.8 (3.9)	66	40.1 (1.7)	11.6 (2.0)^d^	55.2 (7.4)
Haloperidol	40.6 (2.2)	204	35.8 (2.1)	14.9 (2.3)^d^	48.5 (7.8)
Olanzapine	40.2 (2.4)	174	42.3 (1.9)	9.4 (2.0)^d^	58.4 (3.9)
Paliperidone	37.1 (4.5)	43	NA	14.7 (2.1)^d^	52.9 (1.9)
Quetiapine fumarate	43.2 (1.6)	434	37.3 (1.1)	14.4 (1.5)^d^	47.2 (4.6)
Risperidone	48.4 (0.9)	1406	47.2 (1.0)	4.6 (1.5)^d^	57.8 (3.6)
Sulpiride	43.4 (0.9)	1192	43.5 (1.0)	8.3 (1.4)^d^	53.1 (9.8)
Thioridazine hydrochloride	57.1 (9.4)	28	NA^e^	NA^e^	NA^f^
Trifluoperazine hydrochloride	47.2 (4.8)	50	43.6 (1.0)	8.1 (1.9)^d^	58.8 (3.5)
Ziprasidone hydrochloride	43.6 (4.9)	44	43.0 (2.1)	8.8 (2.3)^d^	NA^f^
Zotepine	35.8 (4.1)	49	36.5 (2.0)	15.2 (2.3)^d^	53.3 (5.2)

Abbreviations: ITR, individualized treatment rule; NA, not applicable.

^a^ Refer to the Validation Sample % column in Table 1 for denominators.

^b^ Comparison of expected success rate between patients with medications prescribed according to the ITR (ie, 51.7%) and all patients with a prescription for the medication.

^c^ Refer to the ITR-Recommended Distribution Validation Sample column in Table 1 for the proportions of patients with recommendation by the ITR to receive a prescription for each of the medications.

^d^ Significant difference between observed and expected success rates evaluated using 1-sided α = .05.

^e^ Clothiapine and thioridazine were prescribed to too few patients to allow stable estimates to be made.

^f^ Medication not recommended to anyone by the ITR.

### Key Prescriptive Predictors Used to Build the ITR

Several broad patterns can be seen among the 10 most important prescriptive predictors of the ITR for each of the 15 medications (eTable 4 and eTable 5 in the Supplement). First, age was the only important demographic variable for 6 medications. Second, numerous nonspecific severity measures were important (eg, number of emergency department visits, hospitalizations, and outpatient visits), but these measures involved previous physical disorders (for 12 medications) more than mental disorders (for 5 medications). Third, past use of benzodiazepines was important for 11 medications. Fourth, previous prescription for a mood stabilizer or an antiepileptic drug was important for 10 medications. A prior bipolar diagnosis was not available in the database because such diagnoses were retrospectively recoded as early signs of schizophrenia after a schizophrenia diagnosis was made.

### Medication-Specific Outcomes of ITR-Consistent Prescribing

Medication-specific ITR outcomes were examined in 2 ways among patients with a prescription for nonrecommended medications. First, we estimated the expected increases in treatment success rates if these patients had been given a prescription for their ITR-recommended medication, but this estimation could be done only for 4 medications (amisulpride, aripiprazole, quetiapine, and risperidone) because of the lack of concordance between recommended and prescribed medications (eTable 6 in the Supplement). To carry out the analysis, we needed at least 100 such patients per medication in the validation sample (Table 4). The marginal estimated improvement in the treatment success rate was 3.7% to 9.5% and did not vary significantly across these 4 medications (χ^2^_3_ = 2.2; *P* = .54).

**Table 4.** Estimated Treatment Success Rate of a Prescription for Recommended Medication by Type of Recommended Medication Among Patients With a Prescription for a Nonrecommended Medication^a^

Recommended Medication	Observed Treatment Success Rate		Estimated Success Rate If ITR-Recommended Medication Had Been Prescribed, % (SE)	Difference in Success Rate, % (SE)^c^	No. of Patients Without a Prescription^d^
	% (SE)	No.^b^			
Amisulpride	42.9 (1.0)	1155	46.9 (3.8)	3.7 (3.4)	2693
Aripiprazole	40.5 (0.9)	1156	48.5 (2.8)	5.1 (2.9)^e^	2854
Quetiapine fumarate	39.1 (2.9)	111	49.2 (6.9)	9.5 (4.8)^e^	284
Risperidone	51.2 (2.0)	326	60.9 (4.6)	9.4 (3.1)^e^	637

Abbreviation: ITR, individualized treatment rule.

^a^ Results are presented only for the 4 medications prescribed to at least 100 patients for whom the drug was ITR-recommended. See eTable 5 in the Supplement for the complete distribution of these numbers across medications.

^b^ Number of patients for whom treatment was successful despite not receiving a prescription for their ITR-recommended medication.

^c^ The difference between observed and expected success rates did not vary significantly across ITR-recommended medications (χ^2^_3_ = 2.2; *P* = .54).

^d^ Total number of patients without a prescription for their ITR-recommended medication indicated in the row.

^e^ Significant difference between observed and expected success rates evaluated using 1-sided α = .05.

Second, we estimated how much the treatment success rate would have increased if patients with a prescription for a particular nonrecommended medication had instead received a prescription for their recommended medication. This estimation could be done for 13 of the 15 nonrecommended medications, with the exception of the 2 medications (clothiapine and thioridazine) prescribed to fewer than 100 patients in the validation sample (Table 5). We estimated that the aggregate treatment success rate would have increased differentially across the 13 nonrecommended medications (χ^2^_12_ = 102.2; *P* < .001). Nine of the 13 were associated with significant decrements in estimated treatment success: 3 (chlorpromazine, paliperidone, and zotepine) with greater than 20% success rate, 4 (flupentixol, olanzapine, trifluoperazine, and ziprasidone) with rates in the range of 10% to 20%, and 2 (aripiprazole and sulpiride) with a 5.4% rate. The remaining 4 medications were associated either with nonsignificant decrements in treatment success (amisulpride, 3.8% [SE, 2.4%]; haloperidol, 1.8% [SE, 2.3%]), a nonsignificantly higher success rate than for the recommended medication (risperidone, 1.8% [SE, 1.9%]), and a significantly higher success rate than for the recommended medication (quetiapine, 4.5% [SE, 1.9%]; *Z* = 2.4; *P* = .02).

**Table 5.** Estimated Treatment Success Rate of a Prescription for a Recommended Medication by Type of Nonrecommended Medication Among Patients With a Prescription for a Nonrecommended Medication^a^

Prescribed Medication	Observed Treatment Success Rate		Estimated Success Rate if ITR-Recommended Medication Had Been Prescribed, % (SE)	Difference in Success Rate, % (SE)^c^^,^^d^	No. of Patients Without ITR-Recommended Medication Prescription^d^^,^^e^	Proportional Contribution to Incremental ITR Treatment Success Rate, %
	% (SE)	No.^b^				
Amisulpride	44.3 (2.4)	198	48.1 (0.9)	3.8 (2.4)	447	5.9
Aripiprazole	45.0 (2.6)	171	50.4 (1.5)	5.4 (3.0)^f^	380	7.2
Chlorpromazine	36.9 (4.8)	38	65.0 (3.2)	28.1 (5.3)^f^	103	10.1
Flupentixol	41.8 (3.9)	66	56.0 (1.9)	14.2 (4.2)^f^	158	7.8
Haloperidol	40.5 (2.2)	203	42.3 (1.1)	1.8 (2.3)	501	3.2
Olanzapine	38.8 (2.4)	162	49.1 (1.1)	10.3 (2.5)^f^	417	15.0
Paliperidone	37.6 (4.7)	41	57.9 (2.4)	20.3 (5.1)^f^	109	7.7
Quetiapine	42.4 (1.6)	383	37.9 (0.9)	−4.5 (1.9)^f^	903	−14.2
Risperidone	46.1 (1.0)	1144	44.2 (1.7)	−1.8 (1.9)	2482	−15.6
Sulpiride	43.6 (1.0)	1185	49.0 (0.7)	5.4 (1.0)^f^	2718	51.3
Trifluoperazine	48.0 (5.0)	48	64.4 (2.9)	16.4 (5.6)^f^	100	5.7
Ziprasidone	43.6 (5.0)	44	58.2 (2.4)	14.7 (5.0)^f^	101	5.2
Zotepine	36.7 (4.3)	47	60.4 (1.9)	23.7 (4.6)^f^	128	10.6

Abbreviation: ITR, individualized treatment rule.

^a^ Results are presented only for the 13 medications prescribed to at least 100 patients in the validation sample. See eTable 5 in the Supplement for the complete distribution of these numbers across medications.

^b^ Number of patients for whom treatment was successful despite not receiving a prescription for their ITR-recommended medication.

^c^ Observed and expected success rates varied significantly across prescribed medications (χ^2^_12_ = 102.2; *P* < .001).

^d^ Proportion of the estimated improvement in the treatment success rate of the ITR associated with applying the ITR to each nonrecommended medication was calculated as follows: the product of the difference in success rate and the number of patients without ITR recommended medications divided by the sum of these products across all medications in this table.

^e^ Total number of patients without a prescription for their ITR-recommended medication but instead with a prescription for the medication indicated in the row.

^f^ Significant difference between observed and expected success rates evaluated using 1-sided α = .05.

The medication-specific product of estimated difference in treatment success rate by the number of patients with a prescription for the medication represents the estimated number of patients whose outcome would be improved by using the ITR. Inspection of this proportional distribution shows that the advantage of the ITR was largely associated with correcting nonrecommended prescriptions of either sulpiride (51.3% of total ITR outcome), olanzapine (15.0%), zotepine (10.6%), or chlorpromazine (10.1%). Sulpiride was most important despite the modest reduction in treatment success associated with its nonrecommended use (5.4% [SE, 1.0%]), because it was commonly prescribed (28.4% [SE, 0.9%] of all patients) and only rarely ITR recommended (1.1% [SE, 1.0%] of all patients). Olanzapine, in comparison, had a larger decrement in treatment success associated with its nonrecommended use (10.3% [SE, 2.5%]) but was much less commonly prescribed (4.5% [SE, 1.0%]). Zotepine and chlorpromazine had the largest decrements in treatment success associated with their nonrecommended use (zotepine: 23.7% [SE, 4.6%]; chlorpromazine: 28.1% [SE, 5.3%]) but were seldom prescribed (zotepine: 1.4% [SE, 1.0%]; chlorpromazine: 1.1% [SE, 1.0%]).

## Discussion

To our knowledge, this work is the first machine learning study to develop an ITR for first-episode schizophrenia. We found that a preliminary ITR based on relatively simple patient characteristics had a significantly higher cross-validated estimated treatment success rate (51.7%) compared with the observed population rate (44.5%). The finding that aripiprazole (31.9%) and amisulpride (30.2%) were the most commonly ITR-recommended medications confirmed the broad clinical plausibility of the ITR, given that a network meta-analysis of clinical trials has shown that amisulpride is among the most effective agents for reducing symptom severity and has one of the lowest all-cause discontinuation rates and that aripiprazole is one of the best tolerated drugs overall.^7^ We also found that estimated increases in treatment success rates associated with nonrecommended prescribing of aripiprazole and amisulpride were comparatively small, suggesting that even when prescribed but not recommended, these medications’ treatment success rates were close to those of recommended medications.

Risperidone (30.0%) and sulpride (28.4%) were the most commonly prescribed medications but were ITR recommended for far fewer patients. Risperidone was the third most often ITR recommended but was nonetheless recommended much less often than it was prescribed (11.1%). Sulpride, in comparison, was only rarely recommended (1.1%) and accounted for the largest proportional improvement associated with the ITR.

In 2 cases, the observed treatment success rates for patients who were given a prescription for nonrecommended medications were higher than the success rates predicted if they had received their recommended medications (risperidone and quetiapine). The risperidone difference was not significant, but the quetiapine difference was statistically significant. Under the assumption of no unmeasured confounders, standard practice can never outperform the ITR on the basis of the covariates used to estimate the ITR, unless clinicians combine the variables used to define the ITR in a way that was not discovered in any of the Super Learner classifiers. Thus, the significantly lower performance of the ITR, compared with actual practice, for quetiapine might be owed to clinicians basing their prescribing decisions on considerations that were not included in our model, although the result might instead be owed to chance. The latter possibility could be tested in an independent replication. If the same result was found in a replication, more in-depth investigations of quetiapine prescribing could be conducted to discover the unmeasured prescriptive predictors; in the interim, a revised ITR could be developed that excludes cases in which clinicians would have prescribed quetiapine in the absence of the ITR. In the latter case, we believe that the treatment success rate of the revised ITR would increase.

### Limitations

This study has some limitations. First, and most important, the preliminary ITR was based on an observational design rather than on a randomized controlled design, which could have led to an imbalance in unmeasured baseline covariates that introduced bias into the ITR. However, we explicitly set out to develop an ITR that could be used as an alternative to interaction analysis in future clinical trials to ascertain whether heterogeneous treatment effects might exist rather than used as a definitive clinical decision support tool. Second, the measured predictors were limited, which could have reduced ITR accuracy and led to anomalies, such as those noted with quetiapine. Third, treatment success was defined indirectly rather than by assessing patient symptom severity, functioning, and quality of life, limiting the external validity of the results.

As noted in the introduction, the first limitation is probably inevitable in developing ITRs given the need for samples much larger than those in clinical trials. In other words, the development of explicitly preliminary ITRs in observational studies might be preferable to direct ITR estimation in the necessarily much smaller samples available in controlled trials. The ITRs developed in observational samples can be no more than preliminary until they are subsequently evaluated in controlled trials. The problem of imbalance in observational studies in estimating preliminary ITRs can be addressed by using state-of-the-art estimation methods that adjust for imbalance in observed baseline covariates. Ruling out the existence of unobserved baseline covariates is not possible, which is why it is important to reserve judgment on the value of preliminary ITRs until they are applied in trials, although the latter could include pragmatic trials or stepped-wedge rollouts of the ITRs prior to full implementation.^28^

The second and third limitations may be addressed by analyzing administrative databases that contain richer predictor sets. Text mining could be used to extract structured predictor information from intake clinical notes.^42,43^ These methods could also be used in secondary analyses of existing observational and clinical trials that have richer baseline assessments than the database we used. These limitations will become easier to address in the future as measurement-based care becomes more common in psychiatry and as both intake and tracking data on patient and caregiver symptom reports, clinician ratings, and biomarker assessments become available.^44^

Individualized treatment rules are sensitive to context. For example, hospitalization at the time of first diagnosis of schizophrenia is uncommon in Taiwan, but this is not the case in other countries.^45^ We excluded long-acting injectables from consideration because they were not commonly used in first-episode schizophrenia in Taiwan over the study period, although this trend has subsequently changed and is expected to improve adherence and, through that, treatment response.^46^

In addition, a more nuanced consideration of outcomes might show that treatment success sometimes involves balancing competing risks. For example, a clinician might select a medication for a particular patient in order to avoid metabolic adverse events, but the medication may lead to an increase in bothersome adverse effects that reduce adherence. Scenarios of this sort could be addressed either by (1) defining a single global outcome that assigns values based on the competing effects and developing a single ITR optimized to that global outcome or (2) developing separate ITRs for competing outcomes and allowing clinicians and patients to choose the medications they believe to be optimal in balancing these multiple components of treatment success.

A definitive conclusion to the usefulness of the ITR requires rigorous evaluation in a clinical trial. If the trial result is positive, replication and extension using expanded predictor sets must be conducted. Afterward, clinical implementation of the ITR may be warranted.

## Conclusions

Results of this study suggest that developing a clinically useful ITR to increase 12-month treatment success among patients with first-episode schizophrenia may be possible. Rigorous evaluation in a clinical trial of such an ITR is needed before clinical implementation.

## Notes

Supplement. **eFigure.** Study Population **eTable 1. **The Definitions and Distributions of Baseline Demographic and Clinical Characteristics Used in the Analysis **eTable 2. **Algorithms Included in Super Learner **eTable 3. **Sensitivity Analyses Using Different Definitions of Treatment Success **eTable 4. **The 10 Most Important Predictors in Defining the ITR for Each Antipsychotic Medication, Where Importance Was Defined by Using the Random Forests Variable Importance Method to Perturb One Predictor at a Time to Compare Increases in Mean-Squared Error (MSE) **eTable 5. **Metric Regression Coefficients From Lasso Penalized Regression for the 10 Most Important Predictors in Defining Optimal Treatment Rule by Each Antipsychotic Drug as Determined by Increased MSE in Random Forest Simulations **eTable 6. **The Distributions of Prescribed and ITR-Recommended Medications and Associations Between the Two in the Training Sample
