# Supplementary material for: Development and Validation of a Machine Learning Individualized Treatment Rule in First-Episode Schizophrenia
Source: JAMA Netw Open. 2020 Feb 21;3(2):e1921660. doi: 10.1001/jamanetworkopen.2019.21660 (PMC7043195; doi:10.1001/jamanetworkopen.2019.21660)
Supplement: Supplement. — eFigure. Study Population eTable 1. The Definitions and Distributions of Baseline Demographic and Clinical Characteristics Used in the Analysis eTable 2. Algorithms Included in Super Learner eTable 3. Sensitivity Analyses Using Different Definitions of Treatment Success eTable 4. The 10 Most Important Predictors in Defining the ITR for Each Antipsychotic Medication, Where Importance Was Defined by Using the Random Forests Variable Importance Method to Perturb One Predictor at a Time to Compare Increases in Mean-Squared Error (MSE) eTable 5. Metric Regression Coefficients From Lasso Penalized Regression for the 10 Most Important Predictors in Defining Optimal Treatment Rule by Each Antipsychotic Drug as Determined by Increased MSE in Random Forest Simulations eTable 6. The Distributions of Prescribed and ITR-Recommended Medications and Associations Between the Two in the Training Sample [file jamanetwopen-3-e1921660-s001.pdf]

Supplementary Online Content

Wu C-S, Luedtke AR, Sadikova E, et al. Development and validation of a machine learning individualized treatment rule in first-episode schizophrenia. *JAMA Netw Open.* 2020;3(2):e1921660. doi:10.1001/jamanetworkopen.2019.21660

eFigure. Study Population

eTable 1. The Definitions and Distributions of Baseline Demographic and Clinical Characteristics Used in the Analysis

eTable 2. Algorithms Included in Super Learner

eTable 3. Sensitivity Analyses Using Different Definitions of Treatment Success

eTable 4. The 10 Most Important Predictors in Defining the ITR for Each Antipsychotic Medication, Where Importance was Defined by Using the Random Forests Variable Importance Method to Perturb One Predictor at a Time to Compare Increases in Mean-Squared Error (MSE)

eTable 5. Metric Regression Coefficients From Lasso Penalized Regression for the 10 Most Important Predictors in Defining Optimal Treatment Rule by Each Antipsychotic Drug as Determined by Increased MSE in Random Forest Simulations

eTable 6. The Distributions of Prescribed and ITR-Recommended Medications and Associations Between the Two in the Training Sample

This supplementary material has been provided by the authors to give readers additional information about their work.

eFigure. Study Population

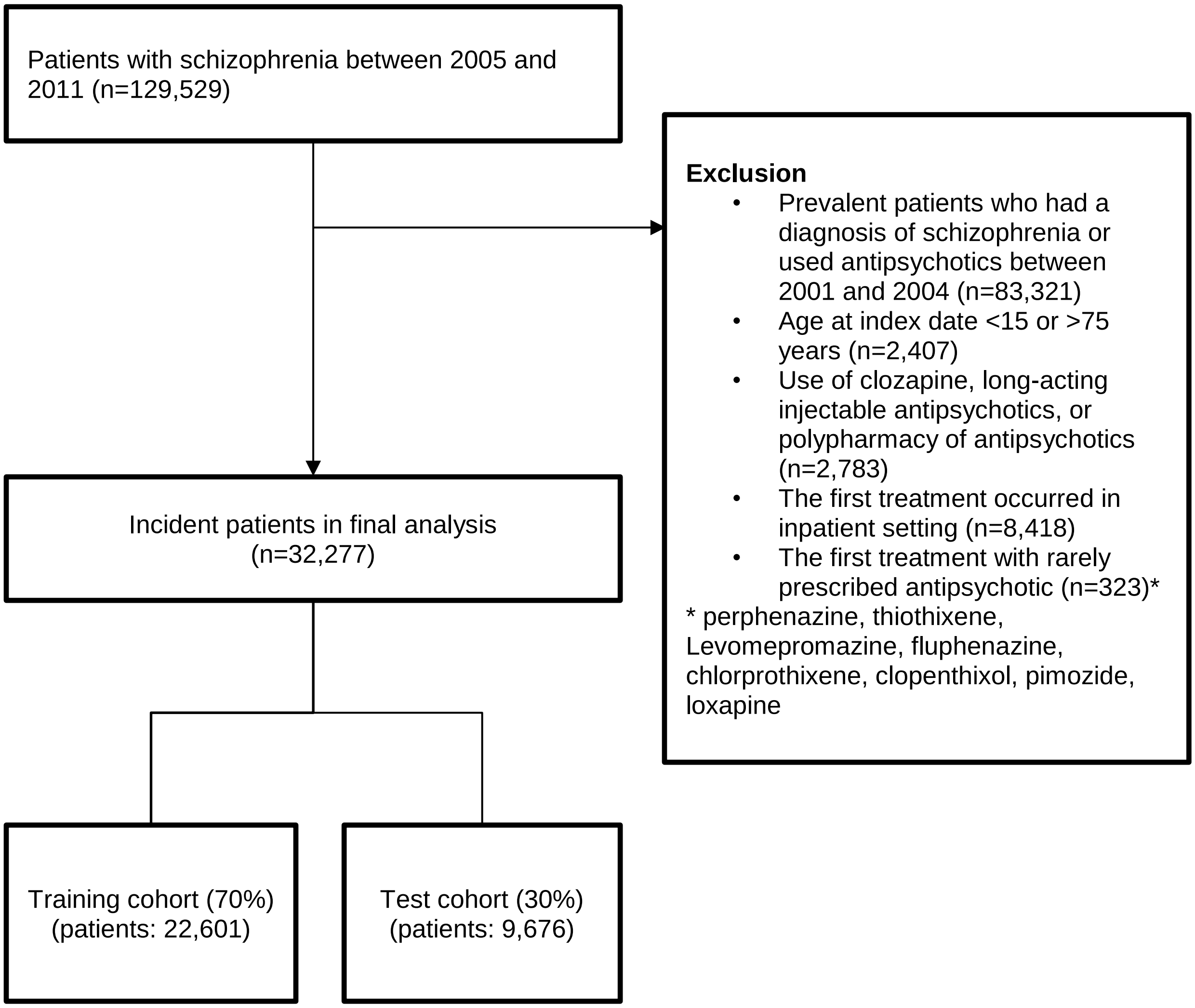

©2020 Wu C-S et al. *JAMA Network Open.*

eTable 1. The Definitions and Distributions of Baseline Demographic and Clinical Characteristics Used in the Analysis

	Training sample		Validation sample dataset		Total sample	
	**Est**	**(SD)**	**Est**	**(SD)**	**Est**	**(SD)**
**I. Demographics at the index date**						
Age (Mean)	36.7	(14.3)	36.6	(14.3)	36.7	(14.3)
Age^2 (Mean)	1,555.3	(1,177.4)	1,541.7	(1,174.8)	1,551.2	(1,176.6)
Male sex (%)	48.6	(0.5)	49.4	(0.5)	48.8	(0.5)
**II. Health system utilization in the past 12 months**						
Number of emergency room visits, psychiatric (%)						
0	92.1	(0.3)	92.3	(0.3)	92.2	(0.3)
1	6.4	(0.2)	6.0	(0.2)	6.3	(0.2)
2 or 3	1.2	(0.1)	1.4	(0.1)	1.3	(0.1)
≥4	0.3	(0.0)	0.3	(0.0)	0.3	(0.0)
Number of outpatient visits, psychiatric (%)						
0-4	91.1	(0.3)	91.3	(0.3)	91.2	(0.3)
5-9	4.8	(0.2)	4.9	(0.2)	4.8	(0.2)
10-14	2.4	(0.2)	2.2	(0.1)	2.4	(0.2)
15-19	0.9	(0.1)	0.9	(0.1)	0.9	(0.1)
20-29	0.6	(0.1)	0.6	(0.1)	0.6	(0.1)
≥30	0.2	(0.0)	0.1	(0.0)	0.2	(0.0)
Number of hospitalizations, psychiatric (%)						
0	99.1	(0.1)	99.0	(0.1)	99.0	(0.1)
1	0.7	(0.1)	0.7	(0.1)	0.7	(0.1)
2	0.1	(0.0)	0.2	(0.0)	0.1	(0.0)
3	0.0	(0.0)	0.1	(0.0)	0.0	(0.0)
≥4	0.1	(0.0)	0.1	(0.0)	0.1	(0.0)
Duration of hospitalization, psychiatric (Mean days)	0.3	(6.2)	0.4	(6.2)	0.3	(6.2)
Duration between index and discharge of last hospitalization, days, non-psychiatric (Mean days)	364.1	(14.1)	364.2	(11.9)	364.1	(13.5)
Number of emergency room visits, non-psychiatric (%)						
0	77.3	(0.4)	77.5	(0.4)	77.4	(0.4)
1	14.7	(0.4)	14.5	(0.4)	14.6	(0.4)
2 or 3	5.9	(0.2)	5.9	(0.2)	5.9	(0.2)
≥4	2.0	(0.1)	2.1	(0.1)	2.1	(0.1)
Number of outpatient visits, non-psychiatric (%)						

©2020 Wu C-S et al. *JAMA Network Open.*

eTable 1 continued. The Definitions and Distributions of Baseline Demographic and Clinical Characteristics Used in the Analysis

	Training sample			Validation sample dataset			Total sample	
	Est	(SD)		Est	(SD)		Est	(SD)
0-4	40.8	(0.5)		41.0	(0.5)		40.9	(0.5)
5-9	18.2	(0.4)		18.8	(0.4)		18.4	(0.4)
10-14	12.0	(0.3)		12.3	(0.3)		12.1	(0.3)
15-19	8.0	(0.3)		8.0	(0.3)		8.0	(0.3)
20-29	9.3	(0.3)		9.2	(0.3)		9.3	(0.3)
≥30	11.7	(0.3)		10.7	(0.3)		11.4	(0.3)
Number of hospitalizations, non-psychiatric (%)								
0	90.0	(0.3)		90.1	(0.3)		90.0	(0.3)
1	6.3	(0.2)		6.2	(0.2)		6.3	(0.2)
2	1.9	(0.1)		1.9	(0.1)		1.9	(0.1)
3	0.8	(0.1)		0.7	(0.1)		0.8	(0.1)
≥4	1.0	(0.1)		1.1	(0.1)		1.0	(0.1)
Duration of hospitalization, non-psychiatric (Mean days)	6.6	(0.2)		6.9	(0.2)		6.7	(0.2)
Duration between index and discharge of last hospitalization, days, non-psychiatric (Mean days)	51.9	(0.5)		53.6	(0.5)		52.4	(0.5)
**III. Clinical diagnoses & comorbid conditions**								
Presence of mood component (%)								
Schizoaffective disorder, at the index date (ICD-9-CM: 295.7)	16.3	(0.4)		16.6	(0.4)		16.4	(0.4)
Major depressive episode, in the past 12 months (ICD-9-CM: 296.2, 296.3)	7.4	(0.3)		7.2	(0.2)		7.4	(0.3)
Minor depression, in the past 12 months (ICD-9-CM: 300.4, 311)	15.7	(0.4)		16.0	(0.4)		15.8	(0.4)
Comorbid psychiatric disorder, in the past 12 months (%)								
Alcohol use disorder (ICD-9-CM: 291, 303.9, 305.0, 357.5, 425.5, 535.3, 571.0-571.3, V11.3)	1.6	(0.1)		1.5	(0.1)		1.6	(0.1)
Anxiety disorder (ICD-9-CM: 300.0-300.3, 300.5-300.9)	21.4	(0.4)		21.5	(0.4)		21.4	(0.4)
Sleep disorder (ICD-9-CM: 307.4, 780.5)	22.9	(0.4)		22.8	(0.5)		22.8	(0.4)
Substance use disorder (ICD-9-CM: 292, 304, 305.2-305.9)	0.8	(0.1)		1.0	(0.1)		0.9	(0.1)
General medical conditions, in the past 12 months (%)								
Acquired immune deficiency syndrome (ICD-9-CM: 042)	0.1	(0.0)		0.1	(0.0)		0.1	(0.0)
Asthma (ICD-9-CM: 493)	3.0	(0.2)		2.8	(0.2)		3.0	(0.2)
Back pain (ICD-9-CM: 721, 722, 723, 724, 739.3,739.4, 846, 847.2)	16.1	(0.4)		16.4	(0.4)		16.2	(0.4)
Cancer (ICD-9-CM: 140-208, 273.0, 273.3, and V10)	1.2	(0.1)		1.2	(0.1)		1.2	(0.1)
Cerebrovascular disease (ICD-9-CM: 362.34, 430-438, 781.4, 784.3, 997.0)	2.6	(0.1)		2.7	(0.2)		2.6	(0.2)

©2020 Wu C-S et al. *JAMA Network Open.*

eTable 1 continued. The Definitions and Distributions of Baseline Demographic and Clinical Characteristics Used in the Analysis

	Training sample			Validation sample dataset			Total sample	
	**Est**	**(SD)**		**Est**	**(SD)**		**Est**	**(SD)**
Chronic kidney disease (ICD-9-CM: 403.x1, 404.x2, 585, 586, V42.0, V45.1, V56.0, V56.8)	0.5	(0.1)		0.6	(0.1)		0.6	(0.1)
Chronic liver disease (ICD-9-CM: 571.2-571.9; 456.0-456.2)	1.1	(0.1)		1.1	(0.1)		1.1	(0.1)
Chronic pulmonary disease (ICD-9-CM: 491, 492, 494, 495, 496)	2.9	(0.2)		2.6	(0.2)		2.8	(0.2)
Congestive heart failure (ICD-9-CM: 402.01, 402.11, 302.91, 425, 428, 429.3)	0.9	(0.1)		1.0	(0.1)		1.0	(0.1)
Coronary heart disease (ICD-9-CM: 410-414)	3.0	(0.2)		2.7	(0.2)		2.9	(0.2)
Diabetes mellitus (ICD-9-CM: 250)	4.6	(0.2)		4.6	(0.2)		4.6	(0.2)
Dyslipidemia (ICD-9-CM: 272)	5.0	(0.2)		4.8	(0.2)		4.9	(0.2)
Fibromyalgia and osteoarthritis (ICD-9-CM: 715, 729.1)	16.4	(0.4)		16.3	(0.4)		16.4	(0.4)
Headache (ICD-9-CM: 307.81, 346, 784.0)	19.1	(0.4)		19.1	(0.4)		19.1	(0.4)
Hemiplegia (ICD-9-CM: 342, 344)	0.5	(0.0)		0.5	(0.1)		0.5	(0.1)
Hypertension (ICD-9-CM: 401-405)	8.7	(0.3)		9.0	(0.3)		8.8	(0.3)
Peptic ulcer disease (ICD-9-CM: 531 – 534)	7.9	(0.3)		7.8	(0.3)		7.8	(0.3)
Peripheral neurological disorder (ICD-9-CM: 351-357)	3.0	(0.2)		3.1	(0.2)		3.0	(0.2)
Peripheral vascular disease (ICD-9-CM: 440, 441.2, 441.4, 441.7, 441.9, 443.1- 443.9, 447.1, 557, 785.4)	0.6	(0.1)		0.5	(0.1)		0.6	(0.1)
Rheumatological disease (ICD-9-CM: 710.0, 710.1, 710.4, 714.0, 714.1, 714.2, 714.81, 725)	0.5	(0.1)		0.7	(0.1)		0.6	(0.1)
**IV. Psychotropic agents**								
Benzodiazepine, concomitant use (%)	65.8	(0.5)		65.5	(0.5)		65.7	(0.5)
Benzodiazepine, days of inpatient use, past 12 months (Mean)	0.5	(3.4)		0.5	(3.5)		0.5	(3.5)
Benzodiazepine, days of outpatient use, past 12 months (Mean)	38.0	(87.4)		35.8	(83.6)		37.4	(86.3)
SNRI, concomitant use (%)	19.0	(0.4)		19.1	(0.4)		19.0	(0.5)
SNRI, days of inpatient use, past 12 months (Mean)	0.1	(1.9)		0.2	(2.4)		0.1	(2.1)
SNRI, days of outpatient use, past 12 months (Mean)	12.1	(45.7)		11.6	(43.2)		11.9	(45.0)
SSRI, concomitant use (%)	3.5	(0.2)		3.5	(0.2)		3.5	(0.2)
SSRI, days of inpatient use, past 12 months (Mean)	0.0	(1.1)		0.0	(0.8)		0.0	(1.0)
SSRI, days of outpatient use, past 12 months (Mean)	2.6	(21.4)		2.4	(20.3)		2.5	(21.0)
TCA, concomitant use (%)	6.0	(0.2)		5.9	(0.2)		6.0	(0.2)
TCA, days of inpatient use, past 12 months (Mean)	0.1	(1.2)		0.1	(1.1)		0.1	(1.2)
TCA, days of outpatient use, past 12 months (Mean)	5.9	(32.5)		6.0	(32.6)		6.0	(32.5)
Other antidepressants, concomitant use (%)	8.7	(0.3)		8.8	(0.3)		8.7	(0.3)
Other antidepressants, days of inpatient use, past 12 months (Mean)	0.1	(1.6)		0.1	(1.7)		0.1	(1.6)

©2020 Wu C-S et al. *JAMA Network Open.*

eTable 1 continued. The Definitions and Distributions of Baseline Demographic and Clinical Characteristics Used in the Analysis

		Training sample			Validation sample dataset			Total sample	
		Est	(SD)		Est	(SD)		Est	(SD)
	Other antidepressants, days of outpatient use, past 12 months (Mean)	8.0	(39.2)		7.7	(37.9)		7.9	(38.8)
	Valproic acid, concomitant use (%)	5.2	(0.2)		5.4	(0.2)		5.3	(0.2)
	Valproic acid, days of inpatient use, past 12 months (Mean)	0.1	(1.2)		0.0	(1.0)		0.0	(1.1)
	Valproic acid, days of outpatient use, past 12 months (Mean)	2.3	(21.7)		2.1	(20.3)		2.2	(21.3)
	Lamotrigine, concomitant use (%)	0.4	(0.1)		0.5	(0.1)		0.5	(0.1)
	Lamotrigine, days of inpatient use, past 12 months (Mean)	0.0	(0.3)		0.0	(0.2)		0.0	(0.3)
	Lamotrigine, days of outpatient use, past 12 months (Mean)	0.4	(9.1)		0.4	(9.5)		0.4	(9.2)
	Carbamazepine, concomitant use (%)	0.8	(0.1)		0.8	(0.1)		0.8	(0.1)
	Carbamazepine, days of inpatient use, past 12 months (Mean)	0.0	(0.8)		0.0	(0.5)		0.0	(0.7)
	Carbamazepine, days of outpatient use, past 12 months (Mean)	1.2	(16.7)		1.0	(14.6)		1.2	(16.1)
	Lithium, concomitant use (%)	1.2	(0.1)		1.1	(0.1)		1.2	(0.1)
	Lithium, days of inpatient use, past 12 months (Mean)	0.0	(0.3)		0.0	(0.1)		0.0	(0.2)
	Lithium, days of outpatient use, past 12 months (Mean)	0.4	(9.9)		0.3	(7.2)		0.4	(9.2)
	Antiepileptic agent,[a] concomitant use (%)	12.9	(0.3)		13.3	(0.3)		13.1	(0.3)
	Antiepileptic agent,[a] inpatient use in the past 12 months, cumulative days (Mean)	0.2	(2.2)		0.2	(2.1)		0.2	(2.2)
	Antiepileptic agent,[a] days of outpatient use, past 12 months (Mean)	8.0	(39.5)		7.1	(36.3)		7.7	(38.6)
V.	**Other medications**								
	ACEI/ARB, concomitant use (%)	2.1	(0.1)		2.2	(0.1)		2.1	(0.1)
	ACEI/ARB, days of inpatient use, past 12 months (Mean)	0.1	(1.4)		0.1	(1.1)		0.1	(1.3)
	ACEI/ARB, days of outpatient use, past 12 months (Mean)	5.7	(33.6)		5.9	(34.8)		5.7	(34.0)
	Anticholinergic, concomitant use (%)	26.1	(0.4)		26.0	(0.4)		26.1	(0.4)
	Anticholinergic, days of inpatient use, past 12 months (Mean)	0.0	(0.5)		0.0	(0.3)		0.0	(0.5)
	Anticholinergic, days of outpatient use, past 12 months (Mean)	1.6	(18.9)		1.6	(18.4)		1.6	(18.8)
	Anti-diabetic agent, concomitant use (%)	2.1	(0.1)		2.2	(0.1)		2.2	(0.1)
	Anti-diabetic agent, days of inpatient use, past 12 months (Mean)	0.1	(1.5)		0.1	(1.5)		0.1	(1.5)
	Anti-diabetic agent, days of outpatient use, past 12 months (Mean)	6.0	(37.9)		6.4	(38.9)		6.1	(38.2)
	Antithrombotic agent, concomitant use (%)	1.6	(0.1)		1.8	(0.1)		1.7	(0.1)
	Antithrombotic agent, days of inpatient use, past 12 months (Mean)	0.1	(1.8)		0.1	(1.5)		0.1	(1.7)
	Antithrombotic agent, days of outpatient use, past 12 months (Mean)	4.8	(32.1)		5.0	(32.6)		4.8	(32.2)
	Beta-blocker, concomitant use (%)	10.0	(0.3)		10.6	(0.3)		10.2	(0.3)
	Beta-blocker, days of inpatient use, past 12 months (Mean)	0.1	(1.8)		0.1	(1.7)		0.1	(1.7)
	Beta-blocker, days of outpatient use, past 12 months (Mean)	11.5	(46.0)		11.6	(46.9)		11.5	(46.3)

©2020 Wu C-S et al. *JAMA Network Open.*

eTable 1 continued. The Definitions and Distributions of Baseline Demographic and Clinical Characteristics Used in the Analysis

	Training sample			Validation sample dataset			Total sample	
	**Est**	**(SD)**		**Est**	**(SD)**		**Est**	**(SD)**
Calcium channel blocker, concomitant use (%)	2.9	(0.2)		3.2	(0.2)		3.0	(0.2)
Calcium channel blocker, days of inpatient use, past 12 months (Mean)	0.2	(2.0)		0.2	(2.0)		0.2	(2.0)
Calcium channel blocker, days of outpatient use, past 12 months (Mean)	7.4	(38.5)		7.9	(41.1)		7.5	(39.3)
Diuretics, concomitant use (%)	1.0	(0.1)		1.1	(0.1)		1.0	(0.1)
Diuretics, days of inpatient use, past 12 months (Mean)	0.1	(1.8)		0.2	(2.0)		0.1	(1.9)
Diuretics, days of outpatient use, past 12 months (Mean)	2.7	(21.9)		2.9	(23.5)		2.7	(22.4)
Lipid lowering agent, concomitant use (%)	1.1	(0.1)		1.0	(0.1)		1.0	(0.1)
Lipid lowering agent, days of inpatient use, past 12 months (Mean)	0.0	(0.9)		0.1	(1.1)		0.0	(0.9)
Lipid lowering agent, days of outpatient use, past 12 months (Mean)	3.2	(23.4)		3.3	(24.6)		3.2	(23.8)
NSAID, concomitant use (%)	6.6	(0.2)		6.1	(0.2)		6.5	(0.2)
NSAID, days of inpatient use, past 12 months (Mean)	0.5	(3.0)		0.4	(2.9)		0.5	(2.9)
NSAID, days of outpatient use, past 12 months (Mean)	13.8	(36.1)		13.3	(35.0)		13.6	(35.8)
(n)	(22,601)			(9,076)			(32,277)	

ACEI/ARB: angiotensin-converting enzyme inhibitor or angiotensin-receptor blocker
NSAID: Nonsteroidal anti-inflammatory drug
SNRI: Serotonin norepinephrine reuptake inhibitor
SSRI: Serotonin selective reuptake inhibitor
TCA: Tricyclic antidepressant
[a]excluding mood stabilizer

©2020 Wu C-S et al. *JAMA Network Open.*

eTable 2. Algorithms Included in Super Learner

Algorithm description	R functions in SuperLearner	Description
Bayesian GLM	SL.bayesglm	• Uses student-t prior distributions to estimate regression coefficients in a generalized linear model setting<br>• Estimates from Bayes GLM are more stable and safeguarded from the problem of complete or quazi-complete separation of points
Generalized additive model	SL.gam	• Multiple regression model where non-parametric functions of the individual predictors are estimated in an additive framework to predict the outcome
Generalized linear model	SL.speedglm	• Traditional parametric logistic regression<br>• Prone to overfit if independent variables are highly collinear<br>• Optimal functional form of independent variables unknown (e.g., linear versus non-linear)
Ridge	SL.glmnet (alpha=0)	• Penalized regression reduces overfit due to collinear independent variables<br>• Ridge regression shrinks coefficients for collinear independent variables *toward* zero, but does not fully-eliminate any independent variable<br>• Elastic net regression allows various penalties where coefficients for collinear independent variables are shrunk *toward* zero (but not to eliminating contributions to the predicted probability) and/or *to* zero (eliminating their contributions to the predicted probability)<br>• Mixing parameter penalty (i.e., alpha) is set somewhere between .01 and .99. Three elastic net algorithms were examined here (mixing parameter penalty set to 0.25, 0.50, and 0.75)<br>• Lasso regression shrinks coefficients for collinear covariate coefficients to zero, eliminating their contributions to the predicted probability
Elastic net	SL.glmnet (alpha=0.25)	
	SL.glmnet (alpha=0.5)	
	SL.glmnet (alpha=0.75)	
LASSO	SL.glmnet (alpha=1)	

©2020 Wu C-S et al. *JAMA Network Open.*

eTable 2 continued. Algorithms Included in Super Learner

Algorithm description	R functions in SuperLearner	Description
Support vector machine	SL.ksvm (kernel = "rbfdot")	• Support vector machines treats each independent variables as dimensions in high dimensional space and attempts to identify the best hyperplane to separate the sample into classes (e.g., cases and non-cases)<br>• Goal is to find the hyperplane with the maximum margin between the two closest points in space<br>• Captures linear associations, but alternate kernels can be used to capture nonlinearities (polynomial and radial basis kernels were used here)
Neural network	SL.nnet	• Feed-forward neural network with a single hidden layer comprising of 2 nodes, used for multinomial log-linear models
Polynomial spline regression	SL.polymars	• Adaptive spline regression flexibly captures interactions and linear and non-linear associations<br>• Linear segments (splines) of varying slopes are connected and smoothed to create piece-wise curves (basis functions)<br>• Final fit is built using a stepwise procedure that selects the optimal combination of basis functions
Random forest	SL.ranger	• Decision tree methods capture interactions and non-linear associations<br>• Independent variables are partitioned (based on values) and stacked to build decision trees and ensemble an aggregate "forest"<br>• Random forests builds numerous trees in bootstrapped samples and generates an aggregate tree by averaging across trees (reducing overfit)
Extreme gradient boosting	SL.xgboost (max_depth=1, shrinkage=0.01)	• Extreme gradient boosting decision tree algorithm. Final predictions are formulated by models sequentially built (using gradient descent algorithm to minimize loss) to resolve residual error made by existing models<br>• Xgboost is faster than most other boosting algorithms and offers improved performance
	SL.xgboost (max_depth=1, shrinkage=0.1)	
	SL.xgboost (max_depth=2, shrinkage=0.01)	
	SL.xgboost (max_depth=2, shrinkage=0.1)	

©2020 Wu C-S et al. *JAMA Network Open.*

**eTable 2 continued.** Algorithms Included in Super Learner				
**Algorithm description**		**R functions in SuperLearner**		**Description**
		SL.xgboost (max_depth=4, shrinkage=0.01)		
		SL.xgboost (max_depth=4, shrinkage=0.1)		

©2020 Wu C-S et al. *JAMA Network Open.*

eTable 3. Sensitivity Analyses Using Different Definitions of Treatment Success

	Treatment success rate[a]							
	Based on ITR		Observed					
	%	(SE)	%	(SE)	Proportional Increase	NNT[b]	z	p-value
**I. Composite outcomes**								
TC + PH + NPH (primary analysis)	51.7	(1.0)	44.5	(0.5)	1.16	13.9	7.1	<.001
TC + PH	54.0	(1.0)	46.4	(0.5)	1.16	13.2	9.6	<.001
TC + PH + NPH + DC3	39.7	(0.8)	28.3	(0.5)	1.40	8.8	17.5	<.001
TC + PH + NPH + DC6	29.6	(0.9)	20.6	(0.4)	1.48	11.1	12.9	<.001
TC + PH + NPH + DC9	24.9	(0.8)	16.8	(0.4)	1.48	12.3	12.9	<.001
**II. Component outcomes**								
TC	57.0	(1.0)	49.5	(0.5)	1.15	13.3	9.5	<.001
PH	86.9	(0.7)	81.7	(0.4)	1.06	19.2	9.2	<.001
NPH	93.9	(0.5)	91.5	(0.3)	1.03	41.7	5.9	<.001
DC3	83.3	(0.8)	81.3	(0.4)	1.02	50.0	2.4	0.017
DC6	73.9	(0.9)	72.9	(0.5)	1.01	100.0	1.4	0.160
DC9	68.9	(0.9)	68.6	(0.5)	1.00	333.3	0.4	0.676

TC=treatment change; PH=psychiatric hospitalization; NPH=non-psychiatric hospitalization; DC3=discontinuation within 3 months, DC6=discontinuation within 6 months; DC9=discontinuation within 9 months

[a]Treatment success rate = 1 – Treatment failure rate.

[b]NNT=Number Needed to Treat (100/difference in treatment success rates)

©2020 Wu C-S et al. *JAMA Network Open.*

eTable 4. The 10 Most Important Predictors in Defining the ITR for Each Antipsychotic Medication, Where Importance was Defined by Using the Random Forests Variable Importance Method to Perturb One Predictor at a Time to Compare Increases in Mean-Squared Error (MSE)[1]

(Entries in the table are MSE x 10e-6)

	Am	Ar	Ch	Cl	Fl	Ha	Ol	Pa	Qu	Ri	Su	Th	Tr	Zi	Zo
**I. Demographic**															
Age	2.9	2.4				0.7		3.4				4.3		1.5	
Age^2	3.1	2.4		0.0		0.6		3.3				4.5		1.7	
Male Sex		1.5	0.5					1.0							
**II. Health system utilization**															
Number of emergency room visits, psychiatric		0.2												1.1	
Number of outpatient visits, psychiatric	1.1				2.0	1.1				0.4					1.8
Number of emergency room visits, non-psychiatric			0.6		1.8		2.0	1.2	1.8					1.0	
Number of hospitalization, non-psychiatric			0.4		0.9		1.2	1.7							
Number of outpatient visits, non-psychiatric	1.6		0.9			0.5	2.0				0.4	2.7	1.5	0.3	
Duration of hospitalization, non-psychiatric (Mean days)								2.1			0.2				1.0
Duration between index and discharge of last hospitalization, days, non-psychiatric (Mean days)								2.2			0.2				0.9
**III. Clinical diagnosis & comorbid conditions**															
Presence of mood component															
Schizoaffective disorder			1.4						2.5			2.7		1.8	
Comorbid psychiatric disorder															
Anxiety disorder									0.9	0.2		0.9			1.7
Sleep disorder			0.2						2.1	0.3					
General medical conditions															
Cancer						0.4									1.7
Cerebrovascular disease		0.4													
Chronic liver disease								0.6							
Diabetes mellitus													2.4		
Dyslipidemia													0.2		
Fibromyalgia and osteoarthritis	1.0										0.5				
Headache					1.3							0.2			
Hypertension				0.1											
Peripheral neurological disorder									1.0						
Peripheral vascular disease						0.4									

©2020 Wu C-S et al. *JAMA Network Open.*

eTable 4 continued. The 10 Most Important Predictors in Defining the ITR for Each Antipsychotic Medication, Where Importance was Defined by Using the Random Forests Variable Importance Method to Perturb One Predictor at a Time to Compare Increases in Mean-Squared Error (MSE)[1]

(Entries in the table are MSE x 10e-6)

	Am	Ar	Ch	Cl	Fl	Ha	Ol	Pa	Qu	Ri	Su	Th	Tr	Zi	Zo
**IV. Psychotropic agents**															
Benzodiazepine, concomitant use		0.5	1.6				1.8		1.1	0.2					
Benzodiazepine, days of inpatient use, past 12 months (Mean)							1.1				0.6				
Benzodiazepine, days of outpatient use, past 12 months (Mean)	1.7	0.2	0.4		1.8	0.6			2.3	0.7	0.2	0.3		0.3	1.6
SNRI, concomitant use						0.5			1.2		0.4				
SNRI, days of outpatient use, past 12 months (Mean)		0.3													
SSRI, concomitant use					1.9	0.5			1.6			0.3		0.8	4.5
SSRI, days of outpatient use, past 12 months (Mean)	0.9				1.5				1.2	0.2		0.4		0.5	
TCA, days of outpatient use, past 12 months (Mean)					1.1										
Other antidepressants, days of outpatient use, past 12 months (Mean)	2.1	0.2													
Valproic acid, concomitant use		0.8	0.4		1.0			1.1						0.4	
Lamotrigine, days of outpatient use, past 12 months (Mean)						0.9									
Carbamazepine, days of outpatient use, past 12 months (Mean)											0.4				
Lithium, concomitant use											0.2				1.7
Antiepileptic agent, concomitant use							2.0			0.2					
Antiepileptic agent, days of outpatient use, past 12 months (Mean)							1.0			0.2	0.4				
Antiepileptic, concomitant use							2.6			0.2					
**V. Other medications**															
ACEI/ARB, concomitant use				0.0			2.5						0.3		
ACEI/ARB, days of outpatient use, past 12 months (Mean)				0.0									0.2		
Anticholinergic, concomitant use															1.4
Anticholinergic, days of outpatient use, past 12 months (Mean)							3.8								1.7
Antidiabetic agent, concomitant use													5.5		
Antidiabetic agent, days of inpatient use, past 12 months (Mean)													0.6		

©2020 Wu C-S et al. *JAMA Network Open.*

eTable 4 continued. The 10 Most Important Predictors in Defining the ITR for Each Antipsychotic Medication, Where Importance was Defined by Using the Random Forests Variable Importance Method to Perturb One Predictor at a Time to Compare Increases in Mean-Squared Error (MSE)[1]

(Entries in the table are MSE x 10e-6)

	Am	Ar	Ch	Cl	Fl	Ha	Ol	Pa	Qu	Ri	Su	Th	Tr	Zi	Zo
Antidiabetic agent, days of outpatient use, past 12 months (Mean)													4.8		
Antithrombotic agent, concomitant use				0.0											
Antithrombotic agent, days of outpatient use, past 12 months (Mean)				0.0											
Beta-blocker, concomitant use				0.6											
Beta-blocker, days of outpatient use, past 12 months (Mean)	1.0			0.1	1.0					0.2					
Calcium channel blocker, concomitant use				0.7											
Calcium channel blocker, days of outpatient use, past 12 months (Mean)				0.2											
Diuretics, days of outpatient use, past 12 months (Mean)	0.9														
Lipid lowering agent, concomitant use													0.3		
Lipid lowering agent, days of outpatient use, past 12 months (Mean)													0.6		
NSAID, days of inpatient use, past 12 months (Mean)								1.9							
NSAID, days of outpatient use, past 12 months (Mean)			0.2									0.6			

ACEI/ARB: angiotensin-converting enzyme inhibitor or angiotensin-receptor blocker
NSAID: Nonsteroidal anti-inflammatory drug
SNRI: Serotonin norepinephrine reuptake inhibitor
SSRI: Serotonin selective reuptake inhibitor
TCA: Tricyclic antidepressant
Am = Amisulpride; Ar = Aripiprazole; Ch = Chlorpromazine; Cl = Clothiapine; Fl = Flupentixol; Ha = Haloperidol; Ol = Olanzapine; Pa = Paliperidone; Qu = Quetiapine; Ri = Risperidone; Su = Sulpiride; Th = Thioridazine; Tr = Trifluoperazine; Zi = Ziprasidone; Zo = Zotepine

[1]Archer KJ, Kimes RV. Empirical characterization of random forest variable importance measures. *Comput Stat Data Anal.* 2008;52(4):2249-2260. doi:10.1016/j.csda.2007.08.015

©2020 Wu C-S et al. *JAMA Network Open.*

eTable 5. Metric Regression Coefficients From Lasso Penalized Regression for the 10 Most Important Predictors in Defining Optimal Treatment Rule by Each Antipsychotic Drug as Determined by Increased MSE in Random Forest Simulations[a]

	Am	Ar	Ch	Cl	Fl	Ha	Ol	Pa	Qu	Ri	Su	Th	Tr	Zi	Zo
**I. Demographic**															
Age	-3.4	-7.8	-4.2	47.3				19.8				-18.4		-9.1	
Age^2	-10.1	-5.7		-43.8		6.6		-34.9						-2.4	
Male Sex	1.7	-4.6	-2.4					-4.3		0.6					
**II. Health system utilization**															
Number of emergency room visits, psychiatric															
0			2.3	-1.3		-1.4								-2.5	
1			-0.8	5.2										4.4	
2 or 3															
≥4			0.9	-0.7						-7.1				-0.3	
Number of outpatient visits, psychiatric															
0-4	2.1		0.1		1.0					0.5					
5-9	0.4		-4.7		-0.3					-0.2					-4.3
10-14			-0.2		-5.5	13.8				-2.6					
15-19	1.4														1.2
20-29	1.7				0.5										
≥30			0.7		1.8	0.2				6.8					1.8
Number of hospitalizations, psychiatric															
0	-5.7					-2.5								-1.9	
1	0.9										-0.8			$7.1x10^{-3}$	
2											3.2				
3	1.7										6.4			0.1	
≥4	-0.9					25.2					-1.2				-0.7
Duration of hospitalization, psychiatric (Mean days)						2.9								6.7	
Number of emergency room visits, non-psychiatric															
0		-1.6	-1.5	0.1	0.8		4.6	-3.7	-2.3					-3.0	1.3
1		0.1			-6.1		-1.0		3.8						-2.1
2 or 3			0.1	-0.2										0.1	
≥4			5.3	-0.1	0.2		0.4		3.5			6.4		0.4	0.7
Number of outpatient visits, non-psychiatric															
0-4	-0.2	-0.1		-0.2	-0.3	-1.6	2.7		0.6	0.2	-1.2	-4.3		-1.1	

©2020 Wu C-S et al. *JAMA Network Open.*

eTable 5 continued. Metric Regression Coefficients From Lasso Penalized Regression for the 10 Most Important Predictors in Defining Optimal Treatment Rule by Each Antipsychotic Drug as Determined by Increased MSE in Random Forest Simulations[a]

	Am	Ar	Ch	Cl	Fl	Ha	Ol	Pa	Qu	Ri	Su	Th	Tr	Zi	Zo
5-9	4.6		-0.5	-0.1		0.1	-0.1		0.1			-0.3			
10-14	0.1	4.2×10^{-3}	3.3				-0.2						6.7		
15-19			-1.4	0.1	0.1	-4.0	-6.9					0.2			
20-29	0.1		-0.4		0.2	0.3			-0.1			3.6			
≥30	-0.5		0.8		-2.2		0.1		-0.9	-0.1	0.9	0.7		8.5×10^{-3}	
Number of hospitalizations, non-psychiatric															
0							-0.1	-4.2			-2.5	-2.1		-0.1	4.3
1			-0.2					0.9			0.4				-0.5
2			2.3		-0.2							0.1			-7.7
3			0.5	0.4	23.1		22.8	-0.3		-1.9		0.1		2.7	
≥4			13.6	1.0	0.5	5.1	0.8	0.3				9.2		2.0	
Duration of hospitalization, non-psychiatric (Mean days)										-2.7					
Duration between index and discharge of last hospitalization, non-psychiatric (Mean days)				-0.7				1.0			0.3				
**III. Clinical diagnosis & comorbid conditions**															
Presence of mood component															
Schizoaffective disorder		0.6	4.7		-1.6				6.1	-0.6		6.4		5.6	4.9
Major depressive episode	-1.3				-1.0					-0.4					-2.2
Minor depression									1.3	-0.3				0.5	-2.1
Comorbid psychiatric disorder															
Alcohol use disorder				0.5											
Anxiety disorder	-0.8		-1.3			-1.5			1.6	-0.4	0.5	2.0			-4.5
Sleep disorder									4.1	-0.6				0.3	
Substance use disorder						1.9									
General medical conditions															
AIDS			7.0											1.2	
Asthma				0.3											
Back pain				-0.3						-0.7				0.5	
Cancer						-9.4									23.0
Cerebrovascular disease		-5.1												-0.2	
Chronic liver disease								13.6			4.3				

©2020 Wu C-S et al. *JAMA Network Open.*

eTable 5 continued. Metric Regression Coefficients From Lasso Penalized Regression for the 10 Most Important Predictors in Defining Optimal Treatment Rule by Each Antipsychotic Drug as Determined by Increased MSE in Random Forest Simulations[a]

	Am	Ar	Ch	Cl	Fl	Ha	Ol	Pa	Qu	Ri	Su	Th	Tr	Zi	Zo
Congestive heart failure				-0.5											
Coronary heart disease				-0.3						-0.8					7.8
Diabetes mellitus	-0.6												4.6		
Dyslipidemia			1.7												
Fibromyalgia and osteoarthritis	-3.3								-0.2	-0.5	2.6				
Headache	-0.6		-1.2	0.2	-4.1					-0.1					
Hemiplegia							27.0								-13.2
Hypertension									2.0						
Peptic ulcer disease					-4.1					-0.5	1.8				
Peripheral neurological disorder									9.8	-0.5					
Peripheral vascular disease					15.9	6.2									
**IV. Psychotropic agents**															
Benzodiazepine, concomitant use		2.2	5.0				4.8		2.4	-0.7					
Benzodiazepine, days of inpatient use, past 12 months (Mean)						1.8					-126.4			0.3	
Benzodiazepine, days of outpatient use, past 12 months (Mean)	-0.5				-0.8				3.6	-1.8				0.1	
SNRI, concomitant use			2.0			4.3			9.1	-1.3	-5.8			0.5	
SNRI, days of outpatient use, past 12 months (Mean)		18.0								-1.9					
SSRI, concomitant use					-4.7	-2.1		2.4	4.4	-0.5				2.9	-2.6
SSRI, days of inpatient use, past 12 months (Mean)															
SSRI, days of outpatient use, past 12 months (Mean)	-9.1		-9.7	0.3	-6.1					-0.4				11.0	-12.6
TCA, concomitant use		1.0							0.3	-0.4					
TCA, days of outpatient use, past 12 months (Mean)				0.3	-14.5					-0.8					
Other antidepressants, concomitant use		0.3		0.2								3.2		0.6	-3.8
Other antidepressants, days of inpatient use, past 12 months (Mean)								50.7							
Other antidepressants, days of outpatient use, past 12 months (Mean)	-17.0	4.0			-6.9					-1.2					
Valproic acid, concomitant use		7.0	6.2		-8.0			9.2	3.1					4.8	
Valproic acid, days of inpatient use, past 12 months (Mean)						3.0									

©2020 Wu C-S et al. *JAMA Network Open.*

eTable 5 continued. Metric Regression Coefficients From Lasso Penalized Regression for the 10 Most Important Predictors in Defining Optimal Treatment Rule by Each Antipsychotic Drug as Determined by Increased MSE in Random Forest Simulations[a]

	Am	Ar	Ch	Cl	Fl	Ha	Ol	Pa	Qu	Ri	Su	Th	Tr	Zi	Zo
Valproic acid, days of outpatient use, past 12 months (Mean)			-5.6												
Lamotrigine, concomitant use		1.6	-7.7							-4.9					
Lamotrigine, days of inpatient use, past 12 months (Mean)											-84.6				
Lamotrigine, days of outpatient use, past 12 months (Mean)						23.5				-4.9				-2.4	
Carbamazepine, concomitant use				0.7											
Carbamazepine, days of outpatient use, past 12 months (Mean)				11.4							15.3				
Lithium, concomitant use		0.9									7.5			0.8	20.1
Lithium, days of inpatient use, past 12 months (Mean)		123.1													
Lithium, days of outpatient use, past 12 months (Mean)		24.4													-10.7
Antiepileptic agent[b] concomitant use							-2.3			-1.0					
Antiepileptic agent[b] days of inpatient use, past 12 months (Mean)	154.9														
Antiepileptic agent[b] days of outpatient use, past 12 months (Mean)	-3.9		-2.6		-0.6		-20.4			-2.0	6.9			-3.4	
Antiepileptic, concomitant use	-3.9		-2.6		-0.6		-20.4			-2.0	6.9			-3.4	
**V. Other medications**															
ACEI/ARB, concomitant use					11.6	157.0	-11.2		2.6						2.7
ACEI/ARB, days of outpatient use, past 12 months (Mean)	-1.6		-14.6	-0.5										-3.2	28.6
Anticholinergic, concomitant use				4.6		0.5	4.5				1.7				
Anticholinergic, days of inpatient use, past 12 months (Mean)	-278.9						158.1								
Anticholinergic, days of outpatient use, past 12 months (Mean)	-12.0			1.3	2.3	8.8	-61.4								
Anti-diabetic agent, concomitant use	-1.0												27.0		
Anti-diabetic agent, days of inpatient use, past 12 months (Mean)										-40.6			49.8		
Anti-diabetic agent, days of outpatient use, past 12 months (Mean)	-0.1												5.1		

©2020 Wu C-S et al. *JAMA Network Open.*

eTable 5 continued. Metric Regression Coefficients From Lasso Penalized Regression for the 10 Most Important Predictors in Defining Optimal Treatment Rule by Each Antipsychotic Drug as Determined by Increased MSE in Random Forest Simulations[a]

	Am	Ar	Ch	Cl	Fl	Ha	Ol	Pa	Qu	Ri	Su	Th	Tr	Zi	Zo
Antithrombotic agent, concomitant use						1.8			5.1×10^{-3}						
Antithrombotic agent, days of outpatient use, past 12 months (Mean)			2.3			1.1				-0.8					
Beta-blocker, concomitant use				7.0					0.4						
Beta-blocker, days of inpatient use, past 12 months (Mean)										-33.6					
Beta-blocker, days of outpatient use, past 12 months (Mean)	-4.0				-11.8					-0.6					
Calcium channel blocker, concomitant use	-1.8					6.1									
Calcium channel blocker, days of outpatient use, past 12 months (Mean)	-13.2										-18.1				
Diuretics, concomitant use				0.6	1.0										
Diuretics, days of inpatient use, past 12 months (Mean)					-303.4					-64.7					
Lipid lowering agent, concomitant use														-0.7	
Lipid lowering agent, days of outpatient use, past 12 months (Mean)			3.2	-1.0						-2.9					
NSAID, concomitant use										-0.9		4.4		0.7	
NSAID, days of inpatient use, past 12 months (Mean)								276.8						1.3	
NSAID, days of outpatient use, past 12 months (Mean)									-2.1	-0.4					

AIDS: Acquired immune deficiency syndrome
ACEI/ARB: angiotensin-converting enzyme inhibitor or angiotensin-receptor blocker
NSAID: Nonsteroidal anti-inflammatory drug
SNRI: Serotonin norepinephrine reuptake inhibitor
SSRI: Serotonin selective reuptake inhibitor
TCA: Tricyclic antidepressant
Am = Amisulpride; Ar = Aripiprazole; Ch = Chlorpromazine; Cl = Clothiapine; Fl = Flupentixol; Ha = Haloperidol; Ol = Olanzapine; Pa = Paliperidone; Qu = Quetiapine; Ri = Risperidone; Su = Sulpiride; Th = Thioridazine; Tr = Trifluoperazine; Zi = Ziprasidone; Zo = Zotepine

[a]The lasso regression models that generated these coefficients contained only the 10 predictors for each outcome in eTable 3a. We present these coefficients to provide some information on relative importance in the effects of the predictors in their metrics rather than in terms of MSE. See eTable 1 for distributions of the predictors. The regression coefficients reported are multiplied by 100. As in eTable 3, the outcome variables are individual-level differences in predicted probabilities of treatment success based on the medication-specific model compared to the model estimated in the total sample (i.e., combined across all medications). See the methods section of the paper and the citations in that section for more details. Note that it is conventional not to report standard errors for lasso regression.

[b]excluding mood stabilizer

©2020 Wu C-S et al. *JAMA Network Open.*

eTable 6. The Distributions of Prescribed and ITR-Recommended Medications and Associations Between the Two in the Training Sample[a]

Prescribed medication	Am	Ar	Ch	Cl	Fl	Ha	Ol	Pa	Qu	Ri	Su	Th	Tr	Zi	Zo	Total
**Amisulpride (Am)**																
Row %	33.7	31.9	1.2	0.0	0.3	0.4	4.0	7.7	2.8	11.7	1.2	0	3.7	0	1.3	
Column %	7.8	7.0	6.7	0.0	3.6	4.8	5.6	6.3	4.9	7.4	7.6		7.4		4.0	
(n)	(227)	(215)	(8)	(0)	(2)	(3)	(27)	(52)	(19)	(79)	(8)		(25)		(9)	(674)
**Aripiprazole (Ar)**																
Row %	35.8	38.1	1.3	0.0	0.3	0.8	2.0	6.0	3.9	6.4	0.6	0	3.3	0	1.5	
Column %	7.5	7.6	6.7	0.0	3.6	8.1	2.5	4.4	6.2	3.6	3.8		5.9		4.0	
(n)	(220)	(234)	(8)	(0)	(2)	(5)	(12)	(37)	(24)	(39)	(4)		(20)		(9)	(614)
**Chlorpromazine (Ch)**																
Row %	20.4	33.0	0.0	0.0	0.0	1.9	2.9	9.7	9.7	15.5	0.0	0	3.9	0	2.9	
Column %	0.7	1.1	0.0	0.0	0.0	3.2	0.6	1.2	2.6	1.5	0.0		1.2		1.4	
(n)	(21)	(34)	(0)	(0)	(0)	(2)	(3)	(10)	(10)	(16)	(0)		(4)		(3)	(103)
**Clothiapine (Cl)**																
Row %	6.4	29.8	8.5	0.0	2.1	2.1	6.4	14.9	10.6	6.4	4.3	0	4.3	0	4.3	
Column %	0.1	0.4	3.4	0.0	1.8	1.6	0.6	0.8	1.3	0.3	1.9		0.6		0.9	
(n)	(3)	(14)	(4)	(0)	(1)	(1)	(3)	(7)	(5)	(3)	(2)		(2)		(2)	(47)
**Flupentixol (Fl)**																
Row %	26.6	29.8	1.7	0.0	0.0	0.0	3.1	6.3	2.5	10.1	0.6	0	3.2	0	10.1	
Column %	1.4	1.5	1.3	0.0	0.0	0.0	9.5	1.2	1.0	1.5	1.0		1.5		7.2	
(n)	(42)	(47)	(2)	(0)	(0)	(0)	(15)	(10)	(4)	(16)	(1)		(5)		(16)	(158)
**Haloperidol (Ha)**																
Row %	25.8	4.5	4.2	0.0	0.8	0.4	9.0	8.2	1.8	16.5	0.8	0	4.2	0	4.2	
Column %	4.4	27.4	1.0	0.0	7.3	3.2	9.4	4.9	2.3	7.8	3.8		6.2		9.5	
(n)	(130)	(138)	(5)	(0)	(4)	(2)	(45)	(41)	(9)	(83)	(4)		(21)		(21)	(503)
**Olanzapine (Ol)**																
Row %	28.9	36.7	5.0	0.2	0.5	0.5	3.7	6.7	3.9	12.5	0.5	0	1.4	0	3.2	
Column %	4.3	5.2	1.4	50.0	3.6	3.2	3.3	3.5	4.4	5.0	1.9		1.8		6.3	
(n)	(125)	(159)	(6)	(1)	(2)	(2)	(16)	(29)	(17)	(54)	(2)		(6)		(14)	(433)
**Paliperidone (Pa)**																
Row %	42.2	25.9	0.0	0.0	0.9	0.0	1.7	6.0	1.7	12.1	0.9	0	5.2	0	3.4	

©2020 Wu C-S et al. *JAMA Network Open.*

eTable 6 continued. The Distributions of Prescribed and ITR-Recommended Medications and Associations Between the Two in the Training Sample[a]

Prescribed medication	Am	Ar	Ch	Cl	Fl	Ha	Ol	Pa	Qu	Ri	Su	Th	Tr	Zi	Zo	Total
Column %	1.7	1.0	0.0	0.0	1.8	0.0	0.4	0.8	0.5	1.3	1.0		1.8		1.8	
(n)	(49)	(30)	(0)	(0)	(1)	(0)	(2)	(7)	(2)	(14)	(1)		(6)		(4)	(116)
**Quetiapine (Qu)**																
Row %	18.3	29.5	2.3	0.0	0.9	1.0	3.1	14.1	10.1	8.9	2.3	0	6.1	0	3.6	
Column %	6.3	9.6	19.3	0.0	16.4	16.1	6.5	17.1	26.2	8.2	21.9		18.0		16.2	
(n)	(184)	(296)	(23)	(0)	(9)	(10)	(31)	(142)	(101)	(88)	(23)		(61)		(36)	(1004)
**Risperidone (Ri)**																
Row %	33.8	30.6	0.9	0.0	0.5	0.5	5.0	6.8	1.7	14.6	0.8	0	2.8	0	1.9	
Column %	33.7	28.8	22.7	50.0	25.4	24.2	30.5	23.7	12.7	39.6	21.9		24.2		25.2	
(n)	(983)	(889)	(27)	(1)	(14)	(15)	(146)	(197)	(49)	(423)	(23)		(82)		(56)	(2905)
**Sulpiride (Su)**																
Row %	30.5	33.4	1.2	0.0	0.6	0.7	32.6	9.6	4.8	7.7	1.1	0	3.4	0	1.4	
Column %	28.7	29.7	27.7	0.0	29.1	32.3	5.7	31.9	34.0	19.8	27.6		27.1		17.1	
(n)	(837)	(918)	(33)	(0)	(16)	(20)	(156)	(265)	(131)	(212)	(29)		(92)		(38)	(2747)
**Thioridazine (Th)**																
Row %	21.4	25.0	7.1	0.0	0.0	0.0	0.0	7.1	7.1	7.1	7.1	0	7.1	0	10.7	
Column %	0.2	0.2	1.7	0.0	0.0	0.0	0.0	0.2	0.5	0.2	1.9		0.6		1.4	
(n)	(6)	(7)	(2)	(0)	(0)	(0)	(0)	(2)	(2)	(2)	(2)		(2)		(3)	(28)
**Trifluoperazine (Tr)**																
Row %	24.5	34.9	0.0	0.0	0.9	0.0	9.4	6.6	0.9	13.2	2.8	0	5.7	0	0.9	
Column %	0.9	1.2	0.0	0.0	1.8	0.0	2.1	0.8	0.3	1.3	2.9		1.8		0.4	
(n)	(26)	(37)	(0)	(0)	(1)	(0)	(10)	(7)	(1)	(14)	(3)		(6)		(1)	(106)
**Ziprasidone (Zi)**																
Row %	28.7	36.6	0.0	0.0	0.0	2.0	3.0	8.9	4.0	7.9	3.0	0	5.0	0	1.0	
Column %	1.0	1.2	0.0	0.0	0.0	3.2	0.6	1.1	1.0	0.8	2.9		1.5		0.4	
(n)	(29)	(37)	(0)	(0)	(0)	(2)	(3)	(9)	(4)	(8)	(3)		(5)		(1)	(101)
**Zotepine (Zo)**																
Row %	27.7	24.1	0.7	0.0	2.2	0.0	7.3	11.7	5.1	13.1	0.0	0	1.5	0	6.6	
Column %	1.3	1.1	0.8	0.0	5.4	0.0	2.1	1.9	1.8	1.7	0.0		0.6		4.0	
(n)	(38)	(33)	(1)	(0)	(3)	(0)	(10)	(16)	(7)	(18)	(0)		(2)		(9)	(137)

©2020 Wu C-S et al. *JAMA Network Open.*

eTable 6 continued. The Distributions of Prescribed and ITR-Recommended Medications and Associations Between the Two in the Training Sample[a]

Prescribed medication	Am	Ar	Ch	Cl	Fl	Ha	Ol	Pa	Qu	Ri	Su	Th	Tr	Zi	Zo	Total
**Total**	(2920)	(3088)	(119)	(2)	(55)	(62)	(479)	(831)	(385)	(1069)	(105)	(0)	(339)	(0)	(222)	(9676)

[a]Each cell includes the number of cases, row %, and column %

©2020 Wu C-S et al. *JAMA Network Open.*
